# Effect of Platelet-Rich Plasma and Bioactive Glass Powder for the Improvement of Rotator Cuff Tendon-to-Bone Healing in a Rabbit Model

**DOI:** 10.3390/ijms151221980

**Published:** 2014-11-28

**Authors:** Yang Wu, Yu Dong, Shiyi Chen, Yunxia Li

**Affiliations:** Department of Sports Medicine, Huashan Hospital, Fudan University, Shanghai 200040, China; E-Mails: wu2013@my.fit.edu (Y.W.); dongyu.dy@163.com (Y.D.); chenshiyi1111@sina.com (S.C.)

**Keywords:** rotator cuff tendon, platelet-rich plasma, bioactive glass

## Abstract

To test the hypothesis that a platelet-rich plasma (PRP) plus bioactive glass (BG) mixture could shorten the tendon-bone healing process in rotator cuff tendon repair, thirty mature male New Zealand white rabbits were randomly divided into three groups, Control, PRP, and PRP + BG. All groups underwent a surgical procedure to establish a rotator cuff tendon healing model. Mechanical examinations and histological assays were taken to verify the adhesion of the tendon-bone. Real-time PCR was adopted to analyze Bone Morphogenetic Protein-2 (BMP-2). The maximum load-to-failure value in mechanical examinations was significantly higher in the PRP + BG group than that in the control group after six weeks (Control 38.73 ± 8.58, PRP 54.49 ± 8.72, PRP + BG 79.15 ± 7.62, *p* < 0.001), but it was not significantly different at 12 weeks (PRP 74.27 ± 7.74, PRP + BG 82.57 ± 6.63, *p* = 0.145). In histological assays, H&E (hematoxylin-eosin) staining showed that the interface between the tendon-bone integration was much sturdier in the PRP + BG group compared to the other two groups at each time point, and more ordered arranged tendon fibers can be seen at 12 weeks. At six weeks, the mRNA expression levels of BMP-2 in the PRP + BG group were higher than those in the other groups (PRP + BG 0.65 ± 0.11, PRP 2.284 ± 0.07, Control 0.12 ± 0.05, *p* < 0.05). However, there was no significant difference in the mRNA expression levels of BMP-2 among the three groups at 12 weeks (*p* = 0.922, 0.067, 0.056). BMP-2 levels in PRP and PRP+BG groups were significantly lower at 12 weeks compared to six weeks (*p* = 0.006, <0.001).We found that the PRP + BG mixture could enhance tendon-bone healing in rotator cuff tendon repair.

## 1. Introduction

Rotator cuff tear (RCT) is a common condition that causes shoulder pain and disability, with more than 50% of the population older than 60 years being afflicted [1]. Most of tears normally require surgical attachment of tendon to its bony insertion. The majority of previous studies focused on suture strength and restoration of the anatomic rotator cuff footprint to strengthen the rotator cuff repair site. However, these methods were still associated with relatively high failure rates because the transitional region was not regenerated. In spite of the improving surgical technique and appropriate postoperative rehabilitation protocol, retear rates following surgical repair of the torn tendon to bone insertion site were still high (20%–90%) [2,3]. Regardless of the different surgical techniques and rehabilitation protocols, the tendon-to-bone integration plays an important role in RCT repair.

Platelet-rich plasma (PRP) is a preparation of autologous plasma that contains a higher platelet concentration, allowing it to deliver a greater concentration of autologous growth factors such as platelet-derived growth factor (PDGF), transforming growth factor beta (TGF-β), insulin-like growth factor (IGF-I) and epithelial growth factor (EGF), that regulate cell proliferation, chemotaxis and differentiation [4,5].

Bioactive glass (BG) is a composite comprised of silicon (Si), calcium (Ca) and traces of other elements —magnesium (Mg) and phosphor (P) [6,7]. Bioactive glass has a notable characteristic—the release of soluble ions from the glass surface [8]. Release of soluble silica from the bioactive glass surface is of great importance. It enhances production of collagen type I. Silicon is also a critical element that binds to glycosaminoglycans, thereby playing a role in cross-linking of the molecule with collagen, preventing their enzymatic degradation and promoting collagen stabilization [9]. With properties such asantibacterial, angiogenesis, and bone-bonding, bioactive glass has been widely used in bone related clinical applications including tooth implant in dental surgery, ossicular reconstruction in otolaryngological surgery, bony restoration in maxillofacial surgery and vertebra prostheses in spinal surgery and so on [10].

The concept “biological solutions to biological and medical problems” [11] is emerging as a new paradigm in medicine leading to the development of novel and more optimized biological preparations that might open new avenues in surgery and the treatment of a wide range of diseases. Increasing work has been focused on biological augmentation recently through the use of biological factors at the repair site to enhance the healing response and reestablish the native tendon insertion site [12].

PRP is widely used in tendon or ligament repair [13], while bioactive glass is often used in orthopedic and dentistry applications [8]. Previously, we found that using a bioactive glass (BG) coating could enhance tendon-bone healing in anterior cruciate ligament (ACL) reconstruction [14]. In this paper, we firstly explored the use of PRP/BG in tendon-bone healing in RCT. Based on the new paradigm, we hypothesize that using a PRP and BG mixture could enhance tendon-bone healing in RCT.

## 2. Results and Discussion

### 2.1. Histologic Results

There was no implant migration or gross infection in all RCT complexes, and all the supraspinati muscles were attached.

**Figure 1:**
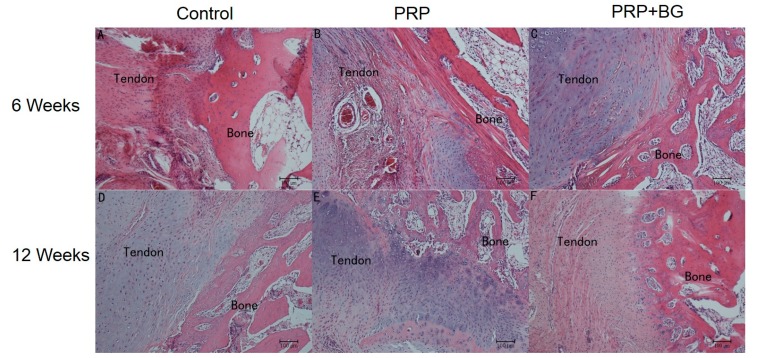
Histological characterization of the control, PRP (platelet-rich plasma), and PRP + BG (bioactive glass) group at 6 and 12 weeks post operation. Hematoxylin-eosin staining, (**A**) Control group 6 weeks; (**B**) PRP group 6 weeks; (**C**) PRP + BG group 6 weeks; (**D**) Control group 12 weeks; (**E**) PRP group 12 weeks; and (**F**) PRP + BG group 12 weeks, magnification ×200).

As shown in Figure 1, tissue cell proliferation was observed in the PRP + BG group at 6 weeks, however, acute inflammatory reactions in the tendon-bone interface was the dominant phenomenon in the control and PRP groups. Compared to the PRP and PRP + BG group, the more distinguished edge was seen in the control group. Furthermore, more hyperchromatic cells and capillary blood vessels can be seen in the PRP group. More collagen can be observed in the PRP + BG group, which showed greater tendon continuity.

At 12 weeks, the interface was more obscure. No obvious acute inflammatory reaction was observed in all groups at this time point. A layer of cellular and fibrous tissue was progressively matured and reorganized as can be seen in PRP + BG group. Compared to the control group, the PRP group presented progressive reestablishment of collagen-fiber continuity between the bone and the tendon, while chondrocytes and osteocytes were more aligned, and more extracellular matrixes were synthesized, in the PRP + BG group.

### 2.2. Mechanical Results

**Figure 2:**
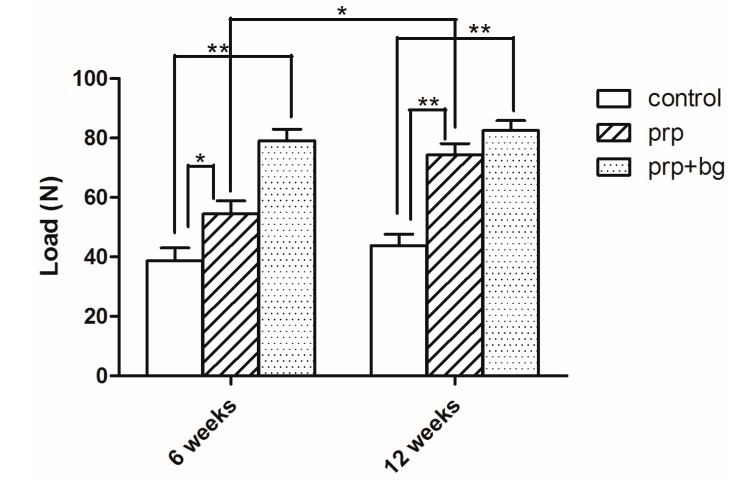
Mechanical examination results at 6 and 12 weeks after surgery. * *p* < 0.05; ** *p* < 0.01.

All specimens failed at the tendon-bone junction. As shown in Figure 2, the mean failure load of the control group was significantly lower than other groups at all time points (*p* < 0.05). As shown in Table 1, the failure load of the PRP group increased significantly compared to the control group at 6 weeks (54.49 ± 8.72; 38.73 ± 8.58, *p* = 0.025, <0.05). While the PRP + BG group showed a significant difference compared to the PRP group (PRP group 54.49 ± 8.72, PRP + BG group 79.15 ± 7.62, *p* < 0.001). Considering the PRP and PRP + BG groups at 12 weeks, we found similar failure loads at this time point with no significant difference (PRP group 74.27 ± 7.74, PRP + BG group 82.57 ± 6.63, *p* = 0.145).

**Table 1:** Multiple comparisons of Mechanical Examination among groups at different times.

Time	Group	Mean (95% CI)	Mean Difference (95% CI)	*p*
6 weeks	Control	38.73 (25.07, 52.38)	-	-
	PRP	54.49 (40.61, 68.37)	15.76 (2.45, 29.07)	0.025 *
	PRP + BG	79.15 (67.03, 91.27)	40.43 (27.11, 53.74)	<0.001 **; 0.002 **
12 weeks	Control	43.83 (31.66, 56.00)	-	-
	PRP	74.27 (61.96, 86.57)	30.43 (18.67, 42.20)	<0.001 **
	PRP + BG	82.57 (72.02, 93.11)	38.73 (26.97, 50.50)	<0.001 **; 0.145

* *p* < 0.05; ** *p* < 0.01.

Table 2 shows that the load of failure in the PRP group increased significantly from 6 to 12 weeks, but no significant difference was observed for the PRP + BG or control group (*p* = 0.015, <0.05).

**Table 2:** *T*-test between different times in 3 groups separately.

Group	Time	Mean(95%CI)	*p*
Control	6 weeks	38.73 (25.07, 52.38)	-
	12 weeks	43.83 (31.66, 56.00)	0.409
PRP	6 week	54.49 (40.61, 68.37)	-
	12 weeks	74.27 (61.96, 86.57)	0.015 *
PRP + BG	6 weeks	79.15 (67.03, 91.27)	-
	12 weeks	82.57 (72.02, 93.11)	0.524

* *p* < 0.05.

### 2.3. RT-PCR Results

**Figure 3:**
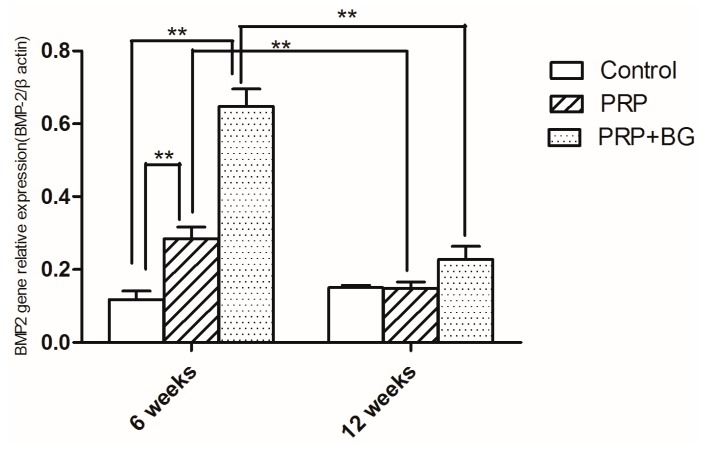
The mRNA expression levels of BMP-2 at 6 and 12 weeks after surgery. * *p* < 0.05; ** *p* < 0.01.

At 6 weeks, the mRNA expression levels of BMP-2 in the PRP + BG group were higher than those in other groups (PRP + BG 0.65 ± 0.11, PRP 0.228 ± 0.07, Control 0.12 ± 0.05, *p* < 0.05), even the PRP group showed higher expression compared to the control group (PRP 2.284 ± 0.07 *versus* Control 0.12 ± 0.05, *p* = 0.007, *p* < 0.01) (Figure 3). However, there were no significant differences in the mRNA expression levels of BMP-2 among the three groups at 12 weeks. The BMP-2 levels in PRP and PRP + BG group had significantly decreased at 12 weeks (*p* < 0.05) (Table 3 and Table 4).

**Table 3:** Multiple comparisons of mRNA levels of BMP-2 among groups at different time point.

Time	Group	Mean (95% CI)	Mean Difference (95% CI)	*p*
6 weeks	Control	0.1180 (0.0544, 0.1816)	-	-
	PRP	0.2840 (0.1946, 0.3734)	0.1660 (0.0554, 0.2766)	0.007 *
	PRP + BG	0.6480 (0.5147, 0.7813)	0.5300 (0.4194, 0.6406)	<0.001 **; *p* < 0.001 **
12 weeks	Control	0.1500 (0.1304, 0.1696)	-	-
	PRP	0.1480 (0.0988, 0.1972)	−0.0020 (−0.0454, 0.0414)	0.922 **
	PRP + BG	0.1900 (0.1479, 0.2321)	0.0400 (−0.0034, 0.0834)	0.067; 0.056

** *p* < 0.01.

**Table 4:** *T*-test between different times in 3 groups separately.

Group	Time	Mean(95%CI)	*p*
Control	6 weeks	0.1180 (0.0544, 0.1816)	-
	12 weeks	0.1500 (0.1304, 0.1696)	0.218
PRP	6 week	0.2840 (0.1946, 0.3734)	-
	12 weeks	0.1480 (0.0988, 0.1972)	0.006 *
PRP + BG	6 weeks	0.6480 (0.5147, 0.7813)	-
	12 weeks	0.1900 (0.1479, 0.2321)	<0.001 *

** *p* < 0.01.

### 2.4. Discussion

The nature of the tendon-to-bone insertion is a functionally graded material that exhibits a gradual transition from soft tissue to hard tissue through a fibrocartilaginous transition region. This unique structure facilitates the effective transfer of load between two materials with vastly different mechanical properties by reducing the potentially damaging stress concentrations that would otherwise form at the interface [15]. Using a rat rotator cuff model, Thomopoulos *et al.* [16] showed that although the structural properties reached two-thirds of their normal levels after eight weeks of healing, the material properties remained weaker than those of the wild-type group. Histologically, a sharp boundary was evident between the soft tissue and the bone. That can also be seen in our study in the control group. Without any further treatment, the suture-only group showed hard tendon-bone healing in terms of structural properties. The edge of the tendon-bone was not that sharp in the PRP + BG group compared to other two groups at each time point in H&E staining indicating a more integrated healing between tendon and bone.

When it comes to material properties, in our study, considering most lesions were gradual onset injury, the low speed rate (1 mm/min) was utilized in mechanical examinations. The mechanical test showed that the PRP + BG mixture could significant improve the max load thus indicating stronger material properties.

The mechanisms and processes of tendon-bone repair are highly complicated. From the perspective of the mechanical role that defines the function of the tendon-to-bone insertion site, Smith *et al.* [17] emphasized the importance of restoring the natural, graded tissue structure during the healing reconstruction; in our study, similar conclusions were reached and in 12 weeks, a distinguishable stratified layer can be seen in the PRP + BG group compared to other groups.

Many factors, environmental, biological, and mechanical, influence the healing process. In particular, the micro-environment of the interface under the influence of postoperative management, significantly affects rotator cuff healing [18]. In view of these facts, multiple solutions, including biologic augmentation [18,19,20], different surgical technique [3,21], and variations in the rehabilitative proposal [22], were adopted to solve the problem.

Furthermore, in patients with large or massive cuff tears, more and more scaffolds have been commonly used based on a variety of synthetic polymers, such as poly-l-lactic acid (PLLA), polylactide-*co*-glycolide (PLGA), and polyurethane, as well as biologic materials, such as collagen and silk. Meanwhile, Biologic material such as extracellular matrix (ECM) and small intestinal submucosa (SIS) are also used in RCT repair [23].

More than 95% of bone is composed mainly of bone mineral and collagen [24]. We also can simplify the tendon-bone healing process as the healing of the integrity of bone, because normal tendon healing is initiated by bone growth into the enthesis [25].

Along with the importance of soluble silica, a previous study suggested that the presence of phosphate ions is also vital for osteoblasts to form calcium phosphate deposition and extracellular mineralized matrix [26]. The majority of studies on gene activation by bioactive glass are related to bone formation, cartilage formation, inflammatory response, vascularity and so on [26]. Researchers testified that the PRP treatment promotes differentiation of tendon stem cells (TSCs) into active tenocytes exhibiting high proliferation rates and collagen production capability [27]. Meanwhile, the PRP can also improve the bone healing potential [20,28,29]. Those characteristic are very important in the process of tendon-bone healing [30].

Bioactive glass can upregulate the expression levels of bone-related genes [26], while PRP can also release bone healing relevant factors at the molecular level [31]. Furthermore, bioactive glass was demonstrated to have anti-inflammatory activity [8], and may pave the way for tendon-bone healing. Additionally, angiogenesis, a property of bioactive glass, can amplify the effect of vascular endothelial growth factor (VEGF).

As important members of the transforming growth factor beta (TGF-beta) family, bone morphogenetic proteins (BMPs) expression is high before significant new bone formation is evident by either radiography or histology assays [32]. Meanwhile, in our study, the expression of BMP-2 had decreased at 12 weeks also observed in Park’s finding that a significant amount of BMP-2 expression may not increase after 4 weeks [33]. In our research, the expression of BMP-2 was increased at 6 weeks, which may be at the downslope of the secretion peak. At 12 weeks, the expression had decreased compared to that of 6 weeks. These findings may suggest that both the PRP group and the PRP + BG group have enhanced expression of BMP-2. Our further studies will focus on earlier time check points to unveil the expression dynamics of BMP-2 and other related genes. But as an early marker, BMP-2 was just evaluated for early bone forming. In further research, other later markers, e.g*.*, matrix proteins, should be taken into consideration.

Our results demonstrate that the PRP plus BG mixture can enhance tendon-bone healing. The mixture can upgrade bone-related gene expression in the tendon-bone healing and improve the strength and maturity of the remodeled tendon-to-bone junction. The mixture is composed of two materials—PRP and BG—and showed the ability to shorten the remolding and healing process in tendon-bone healing.

Dutra *et al.* [34] demonstrated that bioactive glass associated with PRP gave rise to a more mature bone formation. However, some researchers asserted that PRP did not have additional influence on periodontal and bone regeneration [35,36]. Demir *et al.* [37] reported that the addition of PRP to a bioactive glass graft material did not make significant improvements on the treatment of intra-bony defects in clinical studies. In our study, the PRP group showed some potential in tendon-bone healing. When compared to the control group and PRP group, the PRP + BG group presented the potential of shortened tendon-bone healing process in all aspects at 6 weeks. At 12 weeks, the PRP + BG group presented a mature tendon-bone healing compared with others in histologic findings. Meanwhile, the mechanical findings also showed that the PRP + BG group had a strong tendon-bone integration. The expression of BMP-2, however, deceased significantly in the PRP and PRP + BG group at 12 weeks, which indicated that it is an earlier marker of bone formation. Our previous study found that using a bioactive glass (BG) coating could enhance tendon-bone healing in anterior cruciate ligament (ACL) reconstruction. To our knowledge, this study is the first report of a combined used of PRP and BG in an RCT healing model and demonstratesits great therapeutic potential.

We believe that the PRP plus BG treatment could be a good option for patients who have undergone rotator cuff repair. Further studies will focus on elucidating the healing process at the tendon-bone junction. PRP is currently in the developmental stages in some regions, ranging from the laboratory bench top to the patient’s bedside. However, despite these encouraging results, a greater understanding of the functional mechanisms of the PRP/BG mixture needs to be unravelled.

## 3. Experimental Section

Animals were obtained from the Experimental Animal Department of Shanghai Medical College of Fudan University. The animal experiment was approved by the Animal Care and Use Committee of Fudan University (The project identification code: F035, date of approval: January 2014). All rabbits were housed in the animal care laboratory at our university in accordance with the standards established by the National Institutes of Health (Shanghai, China) for the care and use of laboratory animals.

### 3.1. The PRP Preparation

Peripheral blood was obtained immediately before anesthesia, and blood volume (20 mL) was collected from the animals of each group, using EDTA-K3 as anticoagulant [38].

Centrifugal tubes with the blood were centrifuged at 1200 rpm for 10 min at room temperature. The blood was then separated into 3 different parts: red blood cells (at the bottom), platelet-rich plasma (in the middle) and platelet-poor plasma (at the top). The platelet-poor plasma was discarded from the tube and the remaining content was centrifuged again at 1200 rpm for 15 min. Only 2 mL PRP (platelet-rich plasma) was removed and prepared for the experiment [39].

### 3.2. The Mixture Preparation

According to the previous study, the following percentage mixture was used in our research [40]. The 58S BG (60% SiO_2_, 36% CaO, 4% P_2_O_5_) was utilized in this study (provided by Shanghai Research Center of Biomedical Engineering, Shanghai, China). The particle size of 58S glass ranges from 90 to 710 nm. The mixture included 0.02 mg of bioactive glass powder and 2 mL PRP. The mixture was prepared for procedure injection. 

### 3.3. Animal Experiment

Thirty skeletally mature male New Zealand white rabbits (mean weight 3.0 ± 0.2 kg) underwent a surgical procedure to establish a rotator cuff tear. The shoulder was randomly chosen to undergo the rotator cuff tear and performed a repair on rabbit supraspinatus tendons [22]. Anesthetized with 3% pentobarbital (30 mg/kg), the animals were randomly divided in three groups: Control (2 nonabsorbable 3-0 Prolene sutures only (Ethicon, Somerville, NJ, USA)); PRP (the sutured conjunction was sprayed by PRP); PRP + BG (the sutured conjunction was sprayed by PRP + BG). The mixture was sprayed by syringe.

The rabbits were permitted usual cage activity without immobilization until sacrifice at 6 and 12 weeks after surgery.

### 3.4. Histological Analysis

At 6 and 12 weeks, the complexes were prepared for histological analysis of the tendon-bone interface. Immediately after sacrifice, the tendon-bone complex samples were fixed in 10% formalin for 24 h and then embedded un-decalcified in methyl-methacrylate compound, according to established protocols. The samples were sectioned into layers with a thickness of 7 μm perpendicular to the sagittal axis of the humerus tunnel using a Microtome (SM2500, Leica, Wetzlar, Germany).

Serial sections were taken from the area of interest and stained with hematoxylin & eosin (H&E) staining method, and light microscopy (Olympus Co., Osaka, Japan) analysis was performed. Digital images were stored by a DP Manager (Olympus Optical Co., Osaka, Japan).

### 3.5. Mechanical Testing

Immediately after the animals were sacrificed, the integrity of the repair site was inspected, followed by the removal of other tendons and connective tissue (only the supraspinatus tendon was left). Complexes (*n* = 24) were harvested at 6 and 12 weeks after surgery, and prepared for mechanical testing immediately without being frozen. All mechanical testing was performed with an Instron materials testing system machsine (8874, Instron Co., Canton, MA, USA). Fixed firmly and vertically, the Scapula and humerus were performed with an elongation speed of 1 mm/min. The ultimate failure load (N) was measured by the load-deformation curve.

### 3.6. Real-Time Polymerase Chain Reaction

At 6 and 12 weeks, the tendon-bone complex was harvested for real-time polymerase chain reaction (RT-PCR) analysis (*n* = 30). Total RNA from interfacial samples between the host bone and graft were extracted using TRIzol reagent (10296010; Invitrogen, Carlsbad, CA, USA), based on the manufacturer’s instructions. According to the manufacturer’s protocol, cDNA was generated using reverse transcriptase MMLV (D2640A; Takara, Beijing, China). Quantitative PCR was performed with SYBR Premix Ex Taq (DRR041A; Takara, Beijing, China) and then detected using an RT-PCR system (TP800; Takara, Kyoto, Japan). Bone morphogenetic protein-2 (BMP-2) expressions were normalized to that of β-actin.

The primers for RT-PCR were as follows: for β actin, forward 5'-CCA AGG CCA ACC GCG AGA AGATGA-3' and reverse 5'-GCA GCG CGTAGC CCT CGTAGATGG-3'; for BMP-2, forward 5'-GGA ATG ACT GGATTG TGGCT-3' and reverse 5'-TGA GTT CTG TCG GGA CAC AG-3'.

### 3.7. Statistical Analysis

Data were presented as means ± standard deviations (SD).The SPSS for Windows v. 13.0.0 statistical software (SPSS, Inc., Chicago, IL, USA) was taken to perform statistical analysis. The data of the three groups were compared using the one-way ANOVA test, while the LSD (Least-Significant Difference) test was taken to evaluate the differences in each group at the same time point. Comparisons of the various time points of the same group were performed with an independent *t*-test. All *p* values were two sided, and a *p* value of <0.05 was considered to indicate statistical significance.

## 4. Conclusions

The PRP + BG mixture could enhance tendon-bone healing in rotator cuff tendon repair. The mixture of PRP + BG provides a novel means of stimulating tendon-bone healing in RCT and might be useful in stimulating the tendon/bone related factors in tissue-engineering.
